# Studies on the antimicrobial activity and brine shrimp toxicity of *Zeyheria tuberculosa *(Vell.) Bur. (Bignoniaceae) extracts and their main constituents

**DOI:** 10.1186/1476-0711-8-16

**Published:** 2009-05-18

**Authors:** Maria Lysete A Bastos, Maria Raquel F Lima, Lucia M Conserva, Vânia S Andrade, Eliana MM Rocha, Rosangela PL Lemos

**Affiliations:** 1 Escola de Enfermagem e Farmácia, Universidade Federal de Alagoas, 57072-970, Maceió-AL, Brazil; 2 Instituto de Química e Biotecnologia, Universidade Federal de Alagoas, 57072-970, Maceió-AL, Brazil; 3 Instituto de Ciências Biológicas e da Saúde, Universidade Federal de Alagoas, 57010-020, Maceió-AL, Brazil; 4 Instituto do Meio Ambiente do Estado de Alagoas, 57017-320, Maceió-AL, Brazil

## Abstract

**Background:**

Due to the indiscriminate use of antimicrobial drugs, the emergence of human pathogenic microorganisms resistant to major classes of antibiotics has been increased and has caused many clinical problems in the treatment of infectious diseases. Thus, the aim of this study was to evaluate for the first time the *in vitro *antimicrobial activity and brine shrimp lethality of extracts and isolated compounds from *Zeyheria tuberculosa *(Vell.) Bur., a species used in Brazilian folk medicine for treatment of cancer and skin diseases.

**Methods:**

Using the disc diffusion method, bioautography assay and brine shrimp toxicity test (*Artemia salina *Leach), we studied the antimicrobial activity and lethality of extracts and isolated compounds against three microorganisms strains, including Gram-positive (*Staphylococcus aureus*) and Gram-negative (*Pseudomonas aeruginosa*) bacteria and yeasts (*Candida albicans*).

**Results:**

In this study, the extracts inhibited *S. aureus *(8.0 ± 0.0 to 14.0 ± 0.0 mm) and *C. albicans *(15.3 ± 0.68 to 25.6 ± 0.4 mm) growth. In the brine shrimp test, only two of them showed toxic effects (LC_50 _29.55 to 398.05 μg/mL) and some extracts were non-toxic or showed weak lethality (LC_50 _705.02 to > 1000 μg/mL). From these extracts, four flavones [5,6,7,8-tetramethoxyflavone (1), 5,6,7-trimethoxyflavone (2), 4'-hydroxy-5,6,7,8-tetramethoxyflavone (3), and 4'-hydroxy-5,6,7-trimethoxyflavone (4)] were isolated through bioassay-guided fractionation and identified based on the 1D and 2D NMR spectral data. By bioautography assays, compounds 1 [*S. aureus *(16.0 ± 0.0 mm) and *C. albicans *(20.0 ± 0.0 mm)] and 3 [*S. aureus *(10.3 ± 0.6 mm) and *C. albicans *(19.7 ± 0.6 mm)] inhibited both microorganisms while 2 inhibited only *S. aureus *(11.7 ± 0.6 mm). Compound 4 did not restrain the growth of any tested microorganism.

**Conclusion:**

Our results showed that extracts and isolated flavones from *Z. tuberculosa *may be particularly useful against two pathogenic microorganisms, *S. aureus *and *C. albicans*. These results may justify the popular use this species since some fractions tested had antimicrobial activity and others showed significant toxic effects on brine shrimps. However, in order to evaluate possible clinical application in therapy of infectious diseases, further studies about the safety and toxicity of isolated compounds are needed.

## Background

The research into biologically active compounds from natural sources has always been of great interest for scientists looking for new sources of useful drugs against infectious diseases [1]. It is known that the antibiotics commonly used are sometimes associated with adverse effects on the host [2-4]. In recent years, resistance to these drugs by pathogenic bacteria microorganisms has increased and this had caused serious clinical problems in the treatment of infectious diseases [5,6].

The effects of antibiotic resistance have been reflected in the agriculture, food, medical, and pharmaceutical industries. Though pharmacological industries have produced a number of new antibiotics, in the last three decades, yet resistance to these drugs by microorganisms has developed. According to the WHO, medicinal plants would be the best source for obtaining a variety of drugs and a possible way to treat diseases caused by multidrug resistant bacteria [7]. Thus, the *in vitro *antimicrobial activity could provide the needed preliminary observations necessary to select medicinal plants with potentially useful properties for to develop novel antibiotic prototypes [8].

In Brazil, the most common genera of the family Bignoniaceae are *Zeyheria *and *Tabebuia*. They are of economic importance because of its use in the civil construction, furniture manufacturing, paper production, and in urban architecture as ornamental trees [9]. The genus *Zeyheria *comprises about 120 genera and 800 species characterized to be arborous plants which are spread in the tropical regions throughout the world [10]. In Brazil, it comprises only two native species (*Z*. *digitalis *Hoehne & Kuhlm. and *Z. tuberculosa*). From the trunk wood and twigs of *Z. tuberculosa *four naphthoquinones (lapachol, α-dehydrolapachone, α-lapachone, and 4-hydroxy-α-lapachone), two terpenoids (β-amyrin and β-sitosterol) and a lignan (zeyherol) have been isolated [11,12] while from leaves only two flavones (5,6,7-trimethoxyflavone and 5,6,7,8-tetramethoxyflavone) [13].

The aim of this investigation was to evaluate for the first time the *in vitro *antimicrobial activity and brine shrimp lethality of extracts and isolated compounds from *Z*. *tuberculosa*, popularly known as "ipê-preto", against three strains of microorganisms (*S. aureus*, *P. aeruginosa *and *C. albicans*). This plant was selected because in Brazil it has been used for treatment of cancer and skin diseases [11] and there is no antimicrobial study on this species.

## Methods

### General experimental procedures

Hexane, chloroform (CHCl_3_), ethyl acetate (EtOAc), methanol (MeOH), and dimethylsulfoxide (DMSO) analytical grade were purchased from Quimex (F. Maia Indústria e Comércio Ltda, Brazil) or Vetec (Vetec Química Fina Ltda, Rio de Janeiro, Brazil) while Saboraud dextrose agar (SDA), Brain Infusion Heart (BIH) and Mueller Hinton agar (MHA) were purchased from Acumedia Manufacturers Inc. (MI, USA). *Staphylococcus aureus *(ATCC 25923), *Pseudomonas aeruginosa *(ATCC 27853) and *Candida albicans *(ATCC 10231) strains were obtained from the American Type Culture Collection (ATCC, Manassas, VA, USA). Sterile discs of vancomycin, ciprofloxacin and miconazole, gentamicin and 2,3,5-triphenyltetrazolium chloride (TTC) were purchased from Sigma Co (St Louis, MO, USA). Brine shrimp (*Artemia salina *Leach) eggs were bought from Sea Word Comercial Ltda (Maceió, AL, Brazil). Seawater was collected from the Atlantic Ocean, along the North Coast, Alagoas State, Brazil. 1D and 2D NMR spectra were recorded on a Bruker Avance 400 spectrometer (^1^H: 400 MHz and ^13^C: 100 MHz). Column chromatography (CC) were performed on silica gel 60 (70–230 and 230–400 mesh, Merck, Darmstadt, Germany) and Sephadex LH-20 (Pharmacia). Silica gel 60 F_254 _(Merck, Darmstadt, Germany) coated aluminum plates were used for thin-layer chromatography (TLC) and in the bioautography assays.

### Plant material

Stems of *Z. tuberculosa *were collected in June 2005, at the farm Camarão, municipality of Boca da Mata, Alagoas State, Brazil, and identified by Rosangela P. L. Lemos of the Instituto do Meio Ambiente do Estado de Alagoas, where a voucher specimen was deposited (MAC-23816).

### Preparation of the extracts and isolation of the constituents

The air-dried and powdered stems (900 g) were extracted at room temperature with ethanol (EtOH) 90%. The solution was filtered using Whatman N° 1 filter paper under suction and concentrated to dryness at 50°C under reduced pressure. The obtained EtOH extract (7.72 g) was partitioned between hexane, chloroform (CHCl_3_) and hydro-alcoholic (MeOH-H_2_O, 7:3) solution. After that, the methanol (MeOH) was removed under vacuum and the aqueous portion was further extracted with ethyl acetate (EtOAc). Hexane extract (2.9 g) was separated into neutral (1.4 g) and acid (1.5 g) constituents with NaOH 2%. All extracts from these procedures [EtOH, hexane acid (1.4 g), hexane neutral (1.42 g), CHCl_3 _(2.6 g), EtOAc (0.6 g), and MeOH H_2_O (1.5 g)] were evaluated as antimicrobial by disc diffusion method and for lethality to brine shrimp larvae (*Artemia salina *Leach).

The chloroform extract (2.6 g), the most promising in the antimicrobial assays [12.3 ± 0.71 mm (*S*. *aureus*) and 18.3 ± 0.4 mm (*C*. *albicans*)], was further fractionated on silica gel column (70–230 mesh) with hexane-CHCl_3 _1:1 (1.2 g), CHCl_3 _(0.44 g), CHCl_3_-MeOH 1:1 (1.65 g), and MeOH (0.02 g). These fractions were also evaluated and CHCl_3 _fraction (0.44 g), the most active as antimicrobial (14.0 ± 0.0 and 25.6 ± 0.4 mm for *S. aureus *and *C. albicans*, respectively), was fractioned on a silica gel column (70–230 mesh) using hexane containing increasing amounts of EtOAc. Two remaining sub-fractions [53–60 (0.05 g) and 71–74 (0.04 g)] after gel permeation (Sephadex LH-20 with MeOH) and successive recrystallizations from MeOH afforded 1 (25 mg) and **2 **(20 mg), respectively. The CHCl_3_-MeOH 1:1 fraction (1.65 g), active as antimicrobial (8.0 ± 0.0 and 15.3 ± 0.68 mm for *S. aureus *and *C. albicans*, respectively), after successive chromatographic fractionations [silica gel (230–400 mesh, CHCl_3 _containing increasing amounts of MeOH) and Sephadex LH-20 (MeOH)] afforded 3 (5 mg) and 4 (7 mg). These compounds were further evaluated using bioautography assay.

### Antimicrobial assays

In this study, strains of *S. aureus *(ATCC 25923), *P. aeruginosa *(ATCC 27853) and *C. albicans *(ATCC 10231) were used to investigate the antimicrobial potential of the extracts and isolated constituents by disc diffusion method [14]. Stock solutions of the samples [crude extract (50 mg/mL) and fractions (25 mg/mL)] were prepared in chloroform or methanol (HPLC degree, Merck). Further, these solutions were then diluted to give final concentrations ranging from 6.25 to 1000 μg/disc and kept at 4°C prior to use. Sterile discs (6 mm diameter) of vancomycin (30 μg), ciprofloxacin (5 μg), and miconazole (50 μg) were used as positive controls. In order to avoid any effect of the solvent, methanol and chloroform were used as negative controls. The microorganism cultures were grown in Brain Infusion Heart (BIH) liquid medium at 35°C (for bacteria) and 28°C (for yeast), and the microorganisms were kept under refrigeration (4°C) until use. After 24 h (for bacteria) and 48 h (for yeast) of growth, each microorganism, at a concentration of 1.5 × 10^6 ^cells/mL (adjusted to the 0.5 McFarland turbidity standards) [15,16], was inoculated on the surface of Mueller Hinton agar (for bacteria) and Saboraud dextrose agar (for yeast) plates. Sterile filter paper discs (6 mm in diameter) saturated either with extract or fractions were impregnated, in triplicate, with 20 μL of the stock solutions (1000 and 500 μg/disc for extract and fractions, respectively) on surface of each inoculated plate. After holding the plates at room temperature for 1 h to allow diffusion of test samples into the agar, they were incubated [17]. After that, the results were recorded by measuring the zones of growth inhibition around the discs, and presented as the arithmetic average (mean value ± standard deviation). Overall, cultured microorganisms with halos equal to or greater than 7 mm were considered susceptible to samples tested. The data were analyzed using the statistical method of PROBIT analysis with the aid of the SPSS 11.5 computation package (SPSS Inc., Chicago, Illinois, USA).

### Bioautography assay

Isolated compounds (1–4) were evaluated by bioautography technique [18]. An aliquot each sample dissolved in chloroform or methanol or mixture of both [bacteria (10 μg) and yeast (50 μg)] was applied over a chromatosheet of silica gel 60 F_254 _followed by elution with a system of hexane-EtOAc (7:3) and air-drying. Further, a layer of Mueller Hinton agar medium (for bacteria) or Saboraud dextrose agar (for yeast) containing 1.5 × 10^6 ^cells/mL of each microorganism suspension separately was inoculated and sprayed with fresh culture over a developed TLC plate and incubated for 24 h at 35°C (for bacteria) and 48 h at 28°C (for yeast). Each treatment was replicated three-times and gentamicin (10 μg), miconazole (50 μg), solvents and samples-free solutions were used as drugs controls and blank. The inhibition of growth by the compounds on the plates was visualized as white spots against the deep red background after spraying the plates with 2,3,5-triphenyltetrazolium chloride aqueous solution (2.0 mg/mL). The results were recorded by measuring the zones of growth inhibition around the spots and presented as the arithmetic average (mean value ± standard deviation).

### Brine shrimp toxicity assay

The most promising as antimicrobial fractions (hexane acid, hexane-CHCl_3 _1:1, CHCl_3_, and CHCl_3_-MeOH 1:1) were evaluated for lethality to brine shrimp larvae (*A. salina *Leach) according to the procedures described by Meyer et al. [19] and Solis et al. [20]. Brine shrimp eggs were hatched for 48 h in a conical flask containing 300 mL of seawater. The flasks were well aerated with the aid of an air pump and kept in a water bath at 29–30°C. A bright light source was left on and the nauplii hatched within 48 h. The fractions were dissolved in 1% aqueous DMSO to obtain a concentration of 1 mg/mL. These were serially diluted three-times and different concentrations were obtained (1000 to 12.5 μg/mL). An aliquot of each concentration (1 mL) was transferred, in triplicate, into clean sterile universal vials with pipette, and aerated seawater (9 mL) was added. Ten shrimp nauplii were transferred to each vial (30 shrimps per concentration). Thymol 1% aqueous solution [21] and 1% DMSO in seawater were used as positive and negative controls, respectively. After 24 h the numbers of survivors were counted and percentage of death calculated. The concentration that killed 50% of the nauplii (LC_50 _in μg/mL and Confidence Intervals 95%) was determined using the statistical method of PROBIT analysis with the aid of the SPSS 11.5 computation package (SPSS Inc., Chicago, IL, USA) [22]. Criterion of toxicity for fractions was established according to Déciga-campos et al. [23]: LC_50 _values > 1000 μg/mL (non-toxic), ≥ 500 ≤ 1000 μg/mL (weak toxicity) and < 500 μg/mL (toxic).

## Results

### Antimicrobial activity

In this study, no negative controls exhibited antimicrobial activity and compared with positive controls [vancomycin (15.0 ± 0.51 mm) and miconazole (26.0 ± 0.68 mm)], the extracts had significant inhibition against *S*. *aureus *and *C*. *albicans*. In general, medium inhibition zones ranged from 9.0 ± 0.0 to 13.7 ± 0.71 mm and 10.7 ± 0.0 to 25.6 ± 0.4 mm for *S*. *aureus *and *C*. *albicans*, respectively. Among extracts tested, three of the fractions from chromatographic fractionation effectively inhibited the growth of both microorganisms [hexane-CHCl_3 _1:1 (*S. aureus*: 13.7 ± 0.71 mm; *C. albicans*: 21.0 ± 0.7 mm), CHCl_3 _(*S. aureus*: 14.0 ± 0.0 mm; *C. albicans*: 25.6 ± 0.4 mm), and CHCl_3_-MeOH 1:1 (*S. aureus*: 8.0 ± 0.0 mm; *C. albicans*: 15.3 ± 0.7 mm)] while hexane extract (19.0 ± 0.0 mm) and one of its fractions [hexane acid (17.3 ± 0.0 mm)] inhibited only *C*. *albicans*. On the other hand, ethyl acetate and hydro-alcoholic extracts not inhibited the growth of any tested microorganisms. No extract tested inhibited *P. eruginosa *growth.

### Brine shrimp lethality

In the brine shrimp test, among extracts evaluated, two of the fractions [CHCl_3_-MeOH 1:1 (LC50 > 1000 μg/mL) and CHCl_3 _(LC_50 _705.02 μg/mL; CI_95 _422.16–868.03 μg/mL)] were nontoxic or exhibited weak toxicity. On the other hand, hexane acid (LC_50 _29.55 μg/mL; CI_95 _24.67–34.99 μg/mL) and hexane-CHCl_3 _1:1 (LC_50 _398.05 μg/mL; CI_95 _299.14–560.08 μg/mL) fractions showed significant toxic effects. In this assay, the positive control [thymol (1%)] showed significant toxicity (LC_50 _2.9 μg/mL; CI_95 _2.3–3.3 μg/mL).

### Phytochemical investigation

**Figure 1:**
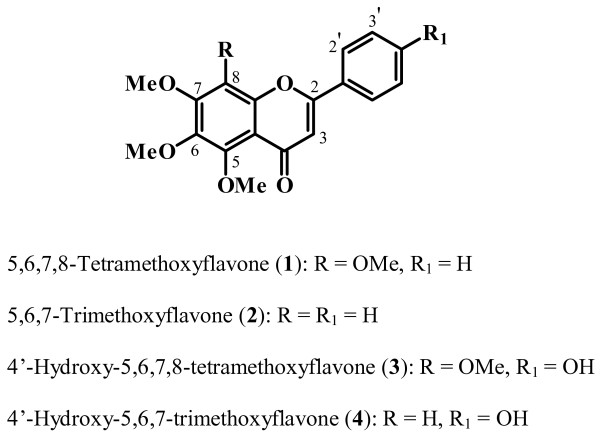
**Chemical structures of isolated compounds**.

From two fractions of promising [CHCl_3 _(*S. aureus*: 14.0 ± 0.0 mm and *C. albicans*: 25.6 ± 0.4 mm) and CHCl_3_-MeOH 1:1 (*S. aureus*: 8.0 ± 0.0 mm and *C. albicans*: 15.3 ± 0.68 mm)], compounds 1–4 were isolated by monitored chromatographic fractionation. Based on the 1D and 2D NMR spectral data (^1^H, ^13^C, DEPT, HMQC and HMBC) as well as comparison with described values, they were identified as 5,6,7,8-tetramethoxyflavone (1) [13,24], 5,6,7-trimethoxyflavone (2) [13,25], 4'-hydroxy-5,6,7,8-tetramethoxyflavone (**3**) [26,27], and 4'-hydroxy-5,6,7-trimethoxyflavone (4) [28] (Figure 1). Among them, compounds 3 and 4 are being described for the first time in the genus *Zeyheria*. When compared to positive controls [gentamicin (15.0 ± 0.25 mm) and miconazole (26.0 ± 0.0 mm)], compounds 1 and 3 inhibited both *S*. *aureus *(1: 16.0 ± 0.0 mm; 3: 10.3 ± 0.6 mm) as *C*. *albicans *(1: 20.0 ± 0.0 mm; 3: 19.7 ± 0.6 mm) while 2 inhibited only *S*. *aureus *(11.7 ± 0.6 mm) and 4 did not restrain the growth of any tested microorganism.

## Discussion

Plants are known to produce some chemical constituents, which are naturally toxic to bacteria and fungi [29]. Traditionally they are used to treat systemic bacterial and yeast infections, as well applied directly on the skin or nails in a plaster form to treat local infections [30]. Despite the significant progress made in molecular biology and synthetic chemistry that can lead to the development of new drugs, plants have been a potential source of bioactive compounds. In this study, extracts from stems of *Z. tuberculosa *showed to be effective against *S. aureus *and *C. albicans*, pathogenic microorganisms known to constitute a significant cause of nosocomial infection and morbidity mainly among immune compromised and severely ill patients [31,32].

Although there are some reports showing that polar extracts inhibited the growth of both Grampositive and Gram-negative bacteria [33,34], in the present study, crude ethanol extract [*S. aureus *(9.0 ± 0.16 mm) and *C*. *albicans *(17.3 ± 0.4 mm)] inhibited only the growth of *S. aureus *(Gram-positive) and *C. albicans *(yeast). This extract was partitioned and only two of remaining extracts (hexane and chloroform) showed significant against *S. aureus *and/or *C. albicans*, and no activity was observed for the most polar extracts (ethyl acetate and hydroalcoholic).

Several studies have shown that brine shrimp assay has been an excellent method for preliminary investigations of toxicity, to screen medicinal plants popularly used for several purposes and for monitoring the isolation a great variety of biologically active compounds [35]. The technique is easily mastered, costs little, and utilizes small amount of test material. Since its introduction [19], this *in vivo *test has been successively employed for bioassay-guide fractionation of active cytotoxic and antitumor agents [36]. Furthermore, a positive correlation between the lethality to brine shrimp and the corresponding oral lethal dose in mice of medicinal plants has been demonstrated by Parra et al. [37]. The results obtained in this study may be support the popular use of *Z. tuberculosa *in Brazil for the treatment of cancer since some fractions showed significant toxic effects on brine shrimps.

From the chloroform and CHCl_3_-MeOH 1:1 fractions, four flavones (1-4) were isolated and also evaluated through bioautography assay against the same microorganisms previously mentioned. Our results showed that the response in terms of susceptibility the response to the microorganism tested varied among them. Compounds 1 and 3, which have four methoxyl groups in the ring A, were actives for *S. aureus *and *C. albicans *while compound 2 (5,6,7-trimethoxyflavone), which lacks a methoxyl group at C-8 of this ring, was active only for *C*. *albicans*. On the other hand, compound 4 (4'-hydroxy-5,6,7-trimethoxyflavone), which lacks a methoxyl group at C-8 and has a hydroxyl group at C-4', in comparison with the similar compound 3, did not affect the growth of the tested microorganisms. These results suggested that the presence of methoxyl group at C-8 can be responsible for the antibacterial activity and that minor structural differences among these flavones influence their antimicrobial activity, bringing new perspectives to studies on the structure-activity relationship of this type of metabolites.

## Conclusion

In Brazil, the species *Z. tuberculosa *has been used in traditional medicine for treatment of cancer and skin diseases. Then, the results obtained in this study may justify the popular use this species since some fractions tested had antimicrobial activity and others showed significant toxic effects on brine shrimps. In addition, our results demonstrated that flavones isolated from this species present *in vitro *antimicrobial activity. However, in order to evaluate possible clinical application in therapy of infectious diseases, further studies about the safety and toxicity of the isolated compounds are needed.

## Competing interests

The authors declare that they have no competing interests.

## Authors' contributions

MLAB was involved in experimental work, designing the study, data acquisition and analysis, literature search, and revising the manuscript. MRFL provided assistance in brine shrimp lethality assay. LMC was responsible for study concept, designing and coordinating the research and writing the manuscript. VSA and EMMR were involved in experimental work, designing the study, data interpretation, and supervised the work. RPLL collected and identified plant material.

All authors read and approved the final manuscript.
